# Extending the Martini 3 Coarse-Grained Force Field to Hyaluronic Acid

**DOI:** 10.1021/acs.jpcb.4c08043

**Published:** 2025-02-24

**Authors:** Valery Lutsyk, Wojciech Plazinski

**Affiliations:** † Jerzy Haber Institute of Catalysis and Surface Chemistry, Polish Academy of Sciences, Niezapominajek 8, 30-239 Krakow, Poland; ‡ Department of Biopharmacy, Medical University of Lublin, Chodzki 4a, 20-093 Lublin, Poland

## Abstract

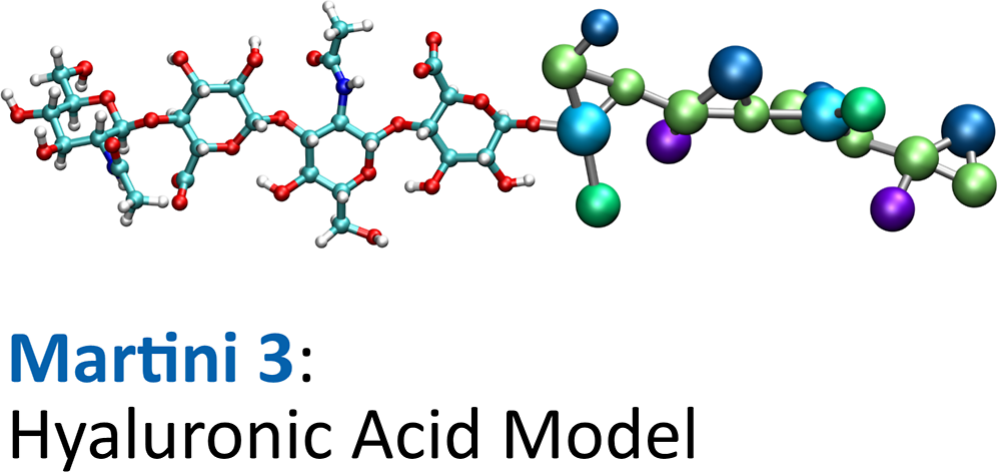

Hyaluronan, also known as hyaluronic acid, is a large glycosaminoglycan composed of repeating disaccharide units. It plays a crucial role in providing structural support, hydration, and facilitating cellular processes in connective tissue, skin, and the extracellular matrix in biological systems. We present a coarse-grained (CG) model of hyaluronic acid (HA) and its constituent residues, *N*-acetyl-d-glucosamine (GlcNAc) and glucuronic acid (GlcA), designed to be compatible with the Martini 3 force field. The model was validated against atomistic molecular dynamics simulations following standard procedures to ensure the accuracy of bonded interactions and, in the case of GlcNAc, the free energies of transfer between octanol and water. For the final HA model, we investigated its properties by simulating the self-assembly of HA chains at varying ion concentrations in solution and comparing the persistence length of single-chain HA with experimental data. We also studied the interactions of HA with lipid bilayers and various HA-binding proteins, demonstrating the ability of the model to accurately reproduce interactions with other biomolecules characteristic of natural biological systems. This extension of the carbohydrate-dedicated branch of the CG Martini 3 force field enables large-scale molecular dynamics simulations of HA-containing systems and contributes to a better understanding of the roles and functions of hyaluronan in natural biomolecular systems.

## 1 Introduction

**Figure 1:**
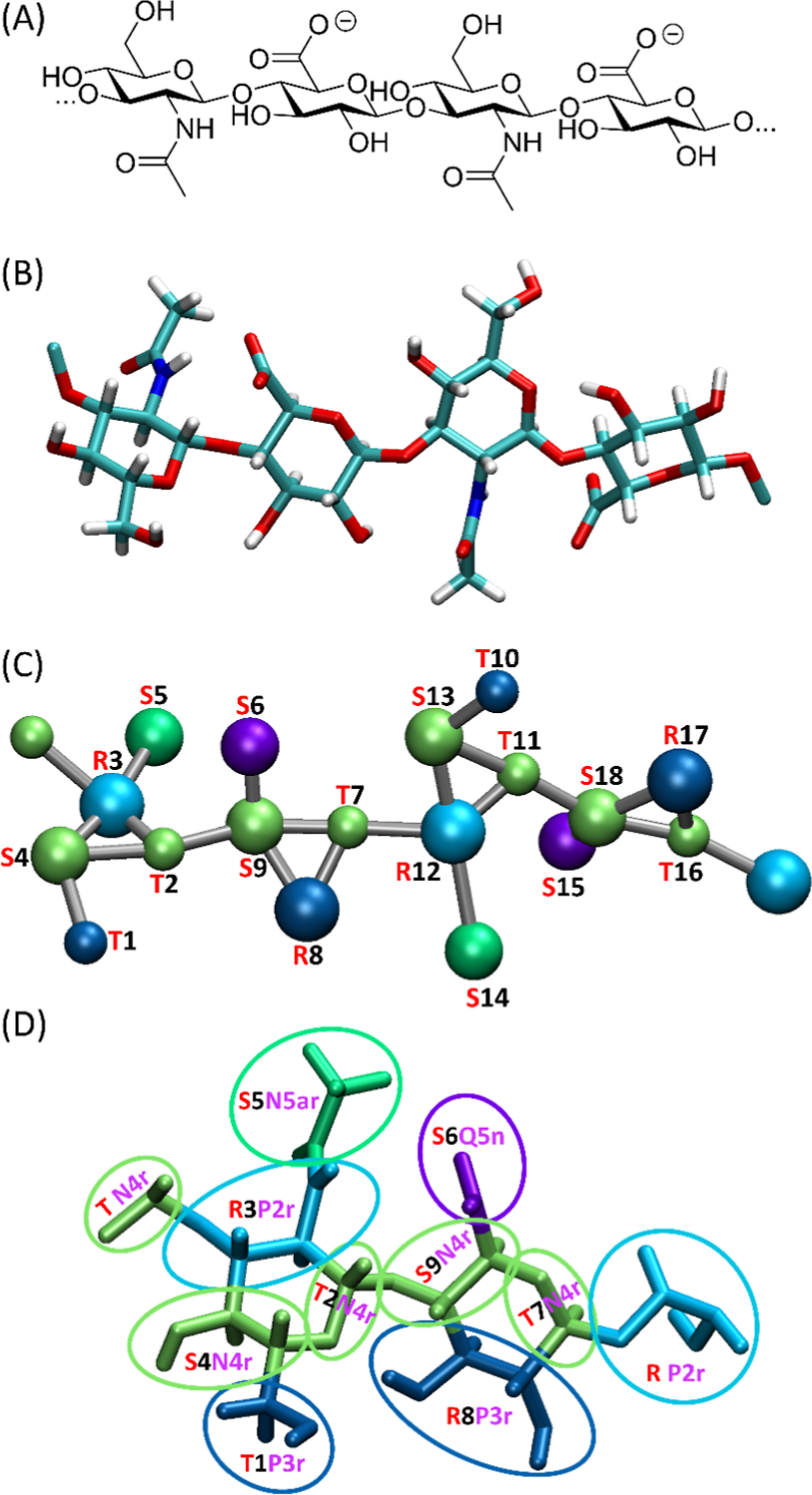
(A) Chemical formula of the HA fragment, i.e., a tetrameric oligosaccharide (deprotonated form). (B) The AA model of the HA tetramer. (C) The CG model of the HA tetramer based on the CG mapping used in the current work. (D) The scheme of AA-to-CG mapping shown for the HA disaccharide (including two **R***P2r* and **T***N4r* beads belonging to the disaccharide-adjacent fragments of the hypothetical longer chain). The bead numbering (1–9) is used to define the CG FF terms (see Tables 2–5).

Hyaluronic acid (HA), also called hyaluronan or hyaluronate, is a linear polysaccharide belonging to the glycosaminoglycan family. It is composed of a repeating disaccharide unit of *N*-acetyl-d-glucosamine (GlcNAc) and glucuronic acid (GlcA) linked by (1 → 3)-β-GlcNAc-(1 → 4)-β-GlcA glycosidic linkages (Figure 1).^1^ Unlike other glycosaminoglycans, HA does not contain sulfate groups and is therefore present in biological systems only in an unfunctionalized form. Despite this fact, HA is involved in many biological processes such as cartilage organization,^2^ maintaining the transparency of the vitreous humor of the eye,^3^ imparting non-Newtonian viscosity to synovial fluid for effective joint lubrication,^4^ and controlling tissue hydration and water transport.^5^ In addition, HA supports essential cellular functions such as adhesion, proliferation and migration.^6^ Interactions with cell surface receptors such as CD44 contribute to its diverse cellular signaling pathways associated with proliferation, differentiation and inflammation.^7^ Its functions also extend to embryonic morphogenesis, wound healing and inflammatory responses.^8^

HA has a relatively rigid random worm-like chain structure^9,10^ and forms a viscoelastic solution at low concentrations.^11^ Its molecular weight can range from 6000 to 8000 kDa,^12^ with a stretched chain length of about 15 μm and a diameter of about 0.5 nm,^13^ although low-molecular-weight polymers of HA have also been found.^14^ Under certain pathological conditions, HA can form large cable-like aggregates that link cells together. These aggregates have specific binding interactions with inactivated mononuclear leukocytes that mediate the inflammatory response.^15^

Due to the complexity of experimental studies on natural polysaccharides, including HA, theoretical methods such as molecular dynamics (MD) simulations are used to understand their biological function^16,17^ and conformational properties in general. However, the large size of polysaccharides in biological systems poses a significant challenge when each atom is treated as a separate interaction center. The coarse-grained (CG) models are a promising alternative to the all-atom (AA) resolution approaches.^18,19^ Depending on the force field (FF) used for CG modeling, each object (interaction center) in the CG FF can describe multiple atoms instead of just one. This allows a significant increase in computational efficiency by reducing the number of objects in the system under study and by increasing the integration step in the iterative MD calculations. One of the most popular families of MD-dedicated CG FFs is the Martini family.^20,21^ The third version of the Martini FF has been released with a wide range of parameters describing various biologically important molecules, including proteins, lipids, phospholipids,^22−25^ some carbohydrates,^26,27^ solvents,^23^ and a number of other organic compounds.^28^

In the current study, we present an extension of the CG Martini 3 force field to the case of HA and its building blocks, i.e., GlcA and GlcNAc. This biologically important polysaccharide has never been parametrized in the current version of Martini FF. The presented model is extensively tested and validated against available experimental and AA MD simulation data. In addition, some new results on the interactions of HA oligomers with lipid bilayers and proteins are reported.

## 2 Theoretical Grounds

### 2.1 General Remarks

The previous version of Martini FF (v. 2) included parameters for carbohydrates,^29^ including HA,^30^ but these parameters (and the whole version of FF) had certain drawbacks, in particular an overestimation of the tendency to self-assembly.^31^ This led to unphysical behavior of the models, especially for carbohydrates.^32^ To overcome these problems, the third version of Martini completely reparametrized the FF parameters, including the mapping rules and the nonbonded parameters, as well as taking into account new data on the physicochemical properties of the molecules.^23^

The currently proposed model of HA and its constituent monosaccharides, GlcNAc and GlcA, is designed to be compatible with the Martini 3 FF,^23^ including the previously proposed carbohydrate extension.^27^ The model includes both the monomeric forms of these saccharides and their glycosylated forms present in HA chains, taking into account the unique structural features and interactions inherent to these molecules. The nonbonded parameters used in this model are based on the CG bead types introduced for the Martini 3 FF^23^ and have been developed using Martini-specific parametrization strategies. These strategies are designed to ensure compatibility with the Martini water model and have been tested in combination with existing Martini models for other biological systems, such as lipid bilayers and proteins. As a result, the current model is fully compatible with Martini 3 FF.

The parametrization was performed incrementally, starting from the two monosaccharides (i.e., GlcNAc and GlcA), through disaccharides, and ending with oligosaccharides. For simplicity, the intermediate attempts required to reach the final parameter set are not discussed. The successive steps of the parametrization procedure followed the typical Martini protocol and included the following points: (1) all-atom-to-coarse-grained mapping, i.e., dividing the realistic molecule into a given number of CG (coarse-grained) beads that best represent the properties of the atomistic model. This step concerned the monomers and was largely based on the mapping proposed in our previous work.^27^ (2) Selection of the type of CG beads, based on the size and physicochemical nature of the individual groups constituting the divided molecule and defining different CG beads. The selection was made from the list of CG beads available in the Martini 3 model, and validation was performed based on, among other things, solvent-accessible surface area (SASA) parameter values from atomistic simulations. Additionally, for the GlcNAc monomer, the log *P* value (strongly dependent on the type of CG beads) was compared with available reference data. (3) Adjustment of bonded parameters for monomers based on reference data from all-atom simulations. (4) Adjustment of bonded parameters for di- and oligosaccharides, including parameters describing the conformation of glycosidic linkages. Also in this case, reference data from all-atom simulations were used. (5) Finally, a series of simulations were performed, focusing primarily on the properties of systems containing HA or its constituent mono- or dimers, but also contributing to the validation of the model (e.g., accurate reproduction of HA-protein binding characteristics or the persistence length of a single HA chain).

This section focuses mainly on the description of the mapping procedure and the functional form of the potentials used in the model. The next Section 3 is devoted to the methodology of simulations and analysis, whereas the following Section 4 describes the final parameters. Finally, Section 5 describes in detail the simulations leading to the final model and the studies of its applicability.

### 2.2 Mapping

Martini 3 mapping uses an approach where 2–4 heavy (i.e., non-hydrogen) atoms are combined into an interaction center called a “bead”. In this way, three different types of CG beads can represent two (tiny bead, T), three (small bead, S), or four (regular bead, R) heavy atoms. The mapping of the current HA model is based on similar concepts as the mapping of the Martini 3 model dedicated to the glucopyranose-containing saccharides previously developed by our team.^27^ This leads to the representation of the GlcNAc molecule by 5 beads and the GlcA molecule by 4 beads (see Figure 1). Thus, the HA dimer can be divided into the following 9 beads.

1. The hydroxymethyl group of GlcNAc (**T1** bead);
2. A ring oxygen, an anomeric carbon and (in the case of the reducing end) the anomeric hydroxyl group of GlcNAc (**T2/S2**);
3. The ethanolamine group of GlcNAc (**R3**);
4. The hydroxyethyl group of GlcNAc (**S4**);
5. The acetyl group bonded to a nitrogen atom of GlcNAc (**S5**);
6. The carboxyl group of GlcA (**S6**);
7. A ring oxygen, an anomeric carbon and (in the case of the reducing end) the anomeric hydroxyl group of GlcA (**T7/S7**);
8. The vicinal diol of GlcA (**R8**);
9. The oxyethyl group of GlcA (**S9**).

The mapping is based on the center-of-geometry (COG) approach, which considers all atoms forming a given CG bead together with aliphatic hydrogens.^23^ The results of the AA MD simulations were used to determine the main parameters of the bonded interactions.

### 2.3 Bonded Interactions

The potential energy term associated with bead–bead bond stretching, which applies to all unique pairs of covalently bonded beads, is given by eq 1
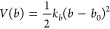
1where *b* is the bond length distance, *b*_0_ its reference value, and *k*_*b*_ is the corresponding force constant.

The potential energy term associated with the bending of the bond angles, which applies to selected triplets of covalently bonded beads, is given by eq 2
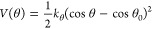
2where θ is the bond angle value, θ_0_ is its reference value, and *k*_θ_ is the corresponding force constant.

In certain cases, especially when the bond–bond angle is close to 180°, a special type of bond angle bending potential (restricted bending potential)^33^ is used to avoid numerical errors and simulation instabilities (eq 3)
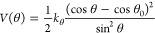
3where all variables are defined as in eq 2.

The potential energy term associated with the deformation of improper dihedral angles, which applies to a subset of atomic quadruplets to control out-of-plane distortions, is given by eq 4
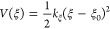
4where ξ is the improper dihedral angle value, ξ_0_ is its reference value, and *k*_ξ_ is the corresponding force constant.

Finally, the potential energy term associated with the torsion of the dihedral angles is given by eq 5
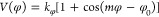
5where φ is the dihedral angle value, *m* is the multiplicity of the term, φ_0_ is the associated phase shift, and *k*_φ_ is the corresponding force constant. This term is applied to only two dihedral angles per HA dimer, as shown in Table 5.

The functions defined by eqs 1–5 are invoked in GROMACS (i.e., the package used for the MD simulations in the current work; see details in further sections) using the following interaction types: eq 1: bonds, type 1; eq 2: angles, type 2; eq 3: angles, type 10; eq 4: dihedrals, type 2; eq 5: dihedrals, type 1.

### 2.4 Nonbonded Interactions

The considered nonbonded interactions are represented by both Lennard–Jones (LJ) and Coulomb potentials. The nonbonded LJ interactions are calculated as a sum over all interacting nonbonded pairs (*i*,*j*) using the following 12/6 interaction function with parameters *C*_12_ and *C*_6_
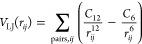
6where *r*_*ij*_ is the distance between the interacting beads. The parameters *C*_12,*ij*_ and *C*_6,*ij*_ depend on the type of beads involved and reflect, e.g., the polarity of the different chemical groups represented by these beads. According to the Martini convention, the first covalent neighbors are excluded from nonbonded interactions.

Since the bead of the GlcA residue containing the carboxyl group is electrically charged, the Coulomb potential is also taken into account in the present model
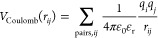
7where *r*_*ij*_ is the distance between the interacting beads, *q*_*ij*_ is the charge of the interacting beads, ε_0_ is the permittivity of the vacuum, and ε_r_ is the relative permittivity of the medium.

## 3 Methods

### 3.1 CG Simulations

The detailed list of systems studied at the CG level is given in Table 1. The initial structures of the saccharides were either drawn manually or generated using the hand-written *python 3* program *carbo2martini3_2.0.py* (an updated version of the program associated with ref (27), included in the Supporting Information). The tool *insane.py* was used to solvate the molecules under consideration, add the number of Na^+^ (or Ca^2+^) and Cl^–^ ions to each system, taking into account their neutral charge and the different ionic strength values, and construct the initial configuration of the lipid bilayer where necessary. The currently proposed FF parameters for the saccharides are given and discussed in detail in the following sections. The other types of molecules (lipids and proteins) were modeled using the Martini 3.0 parameters and prepared using the tools available on the web (*martinize2* and *insane.py*). The simulations were carried out with the GROMACS 2016.4, 2022, or 2023 packages,^34^ under periodic boundary conditions and in the isothermal–isobaric ensemble. The temperature was kept close to the reference value (310 K for the protein and lipid-containing systems and 298 K elsewhere) using the V-rescale thermostat,^35^ while for the constant pressure (1 bar) the Parrinello–Rahman barostat^36^ with a relaxation time of 40 ps was used. Either semi-isotropic (bilayer-containing systems) or isotropic (remaining systems) pressure scaling was applied. The equations of motion were integrated with a time step of 10 (HA-containing, protein and lipid bilayer systems) or 30 fs (monosaccharide systems only) using the leapfrog scheme.^37^ The translational center-of-mass motion was removed separately for the solute and the solvent at each time step. The Martini 3 water model was used.^23^ van der Waals interactions (LJ potentials) are set to zero beyond the 1.1 nm cutoff. For Coulomb interactions, the reaction field approach was used with a cutoff of 1.1 nm and ε_r_ = 15. The details of the MD parameters were kept according to the example *mdp* files deposited on the *cgmartini.nl* web site. The production simulations were performed for a duration of 490–30,000 ns (depending on the system; see Table 1), and the data were saved to the trajectory every 5–50 ps.

**Table 1:** CG Systems Considered in the Present Study^a^

purpose	composition	no. of solvent molecules	box vector lengths [nm^3^]	simulation time [ns]	remarks
conformational properties, SASA	GlcNAc monomer	693 + 8 Na^+^ + 8 Cl^–^	4 × 4 × 4	500	unbiased MD
	GlcA monomer	684 + 1 Na^+^	4 × 4 × 4	500	unbiased MD
	HA dimer	673 + 15 Na^+^ + 14 Cl^–^	4 × 4 × 4	500	unbiased MD
	HA 4-mer	1236 + 27 Na^+^ + 25 Cl^–^	5 × 5 × 5	500	unbiased MD
	HA 8-mer	2023 + 47 Na^+^ + 43 Cl^–^	6 × 6 × 6	500	unbiased MD
	HA 20-mer	9909 + 519 Na^+^ + 509 Cl^–^	14 × 14 × 14	500	unbiased MD
log *P*	GlcNAc monomer	1317	5.4 × 5.4 × 5.4	15 per TI window	TI, various nonbonded parameters
	GlcNAc monomer	439 (octanol)	4.8 × 4.8 × 4.8	15 per TI window	TI, octanol solvent, various nonbonded parameters
aggregation properties, influence of different ion concentrations	25 HA 8-mers	27,503 + 303 Na^+^ + 203 Cl^–^	15 × 15 × 15	5360	unbiased MD
	25 HA 8-mers	26,590 + 706 Na^+^ + 606 Cl^–^	15 × 15 × 15	5410	unbiased MD
	25 HA 8-mers	25,349 + 1307 Na^+^ + 1207 Cl^–^	15 × 15 × 15	4310	unbiased MD
	25 HA 8-mers	27,514 + 152 Ca^2+^ + 204 Cl^–^	15 × 15 × 15	5590	unbiased MD
	25 HA 8-mers	26,878 + 355 Ca^2+^ + 610 Cl^–^	15 × 15 × 15	5520	unbiased MD
	25 HA 8-mers	25,995 + 660 Ca^2+^ + 1220 Cl^–^	15 × 15 × 15	3080	unbiased MD
aggregation properties	4 HA 100-mers	516,614 + 5687 Na^+^ + 5487 Cl^–^	40 × 40 × 40	10,000	unbiased MD
	8 HA 100-mers	513,163 + 5649 Na^+^ + 5487 Cl^–^	40 × 40 × 40	10,000	unbiased MD
	12 HA 100-mers	509,874 + 5613 Na^+^ + 5013 Cl^–^	40 × 40 × 40	10,000	unbiased MD
	24 HA 100-mers	499,787 + 5502 Na^+^ + 4302 Cl^–^	40 × 40 × 40	1510	unbiased MD
persistence length calculation, influence of different ion concentrations	HA 100-mer	530,467 + 50 Na^+^	40 × 40 × 40	5000	unbiased MD
	HA 100-mer	529,657 + 434 Na^+^ + 384 Cl^–^	40 × 40 × 40	5000	unbiased MD
	HA 100-mer	526,613 + 1971 Na^+^ + 1921 Cl^–^	40 × 40 × 40	5000	unbiased MD
	HA 100-mer	522,737 + 3889 Na^+^ + 3839 Cl^–^	40 × 40 × 40	5000	unbiased MD
	HA 100-mer	518,948 + 5806 Na^+^ + 5756 Cl^–^	40 × 40 × 40	5000	unbiased MD
	HA 100-mer	515,112 + 7721 Na^+^ + 7671 Cl^–^	40 × 40 × 40	5000	unbiased MD
interactions with lipid bilayer	134 POPC + 160 POPE + 86 POPS + 262 cholesterol molecules	26,007 + 200 Na^+^ + 28 Cl^–^	15 × 15 × 18	10,000	unbiased MD
	134 POPC + 160 POPE + 86 POPS + 262 cholesterol molecules +20 HA dimers	25,537 + 143 Na^+^ + 37 Cl^–^	15 × 15 × 18	4410	unbiased MD
	134 POPC + 160 POPE + 86 POPS + 262 cholesterol molecules + HA octamer	25,933 + 127 Na^+^ + 37 Cl^–^	15 × 15 × 18	2510	unbiased MD
interactions with proteins	protein (PDB:2JCR) + HA 8-mer	4252 + 100 Na^+^ + 91 Cl^–^	8 × 8 × 8	30,000	unbiased MD
	protein (PDB:1LXK) + HA 4-mer	21,888 + 503 Na^+^ + 487 Cl^–^	14 × 14 × 14	30,000	unbiased MD
	protein (PDB:6SYV) + 10 GlcA monomers	3678 + 100 Na^+^ + 84 Cl^–^	8 × 8 × 8	490	unbiased MD

a Unless otherwise noted, the solvent was Martini 3 water, where 1 CG bead = 4 water molecules.

The solvent-accessible surface area (SASA) parameter was calculated using the double cubic lattice method,^38^ which is the default procedure implemented in the GROMACS package (the *gmx sasa* routine). The atomic radii values were 0.191 nm (***T*** beads), 0.230 nm (***S*** beads), and 0.264 nm (***R*** beads), which is consistent with the methodology used in the original Martini 3 paper.^23^

Log *P* values were calculated as the Gibbs free energy difference corresponding to the transfer of the saccharide molecule from water to *n*-octanol. Calculations were performed for the GlcNAc monomer. To construct the thermodynamic cycle, the GlcNAc molecule was decoupled from both water and *n*-octanol solvents. The decoupling of the GlcNAc molecule from both types of systems was performed by scaling down to zero all nonbonded interactions involving carbohydrate atoms in a stepwise manner as a function of a coupling parameter λ. The associated free energy changes were calculated using the Bennett acceptance ratio (BAR) method^39^ implemented in the GROMACS *gmx bar* subroutine, including the error estimation determined by using the default criteria. The 21 equally spaced thermodynamic integration (TI) λ points were accepted and data from the equilibrated systems were collected every 1 ps for a duration of 15 ns in each λ window. A soft core function was used for the van der Waals interactions to avoid energy singularities.

For the protein-containing systems, the proteins CD44 (PDB: 2JCR), SpnHL (PDB: 1LXK), and OtCE15A (PDB: 6SYV) were transformed from AA to CG models using the *martinize2* tool, excluding all nonprotein residues and with *-elastic*, *-cys auto*, and other default options. Additionally, the elastic bond force constant for the CD44 protein was set to 1000 kJ/mol/nm^2^ (*-ef 1000*) and the His408 residue of the OtCE15A protein was protonated (*-mutate A-HIS408:HSP*). For the CD44 and SpnHL protein systems, 1 HA oligosaccharide and for the OtCE15A protein system, 10 GlcA monomers were initially randomly positioned away from the proteins. After solvation, energy minimization and equilibration, unbiased MD simulations of these systems were performed for up to 30 μs.

### 3.2 Calculation of Persistence Length

One of the parameters of polymers that theoretically does not depend on the chain length, and therefore is a good characterization of the natural elasticity of the chain, is the persistence length. This property can be determined in several ways. According to the Kratky–Porod worm-like chain (WLC) model,^9^ the persistence length (*l*_p_) can be related to the value of the end-to-end (*e*2*e*) parameter, while Benoit and Doty^40^ derived a similar relationship by linking the values of *l*_p_ and the radius of gyration (*R*_g_). In the case of polysaccharides, these two relationships predict nearly the same values of *l*_p_ for the same systems.^41^ Moreover, their predictions are in good agreement with those of the method used in our recent work,^41^ i.e., the approach based on the autocorrelation functions. Let us define the autocorrelation function *C*(*n*) of two bond vectors (*a*_*i*_,*a*_*i*+*n*_) separated by *n* bonds, as
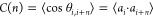
8

Then, the *l*_p_ value can be obtained by fitting the following exponentially decaying function
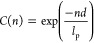
9where *d* is the average residue-to-residue bond length. The values of *C*(*n*) can be extracted from the MD trajectory using eq 8.

This method, based on the best fit of the *l*_p_ value using eq 9 and the data from the MD trajectory transformed using eq 8, was applied to the input data from MD simulations of HA chains. The contour length was assumed to be equal to *d* multiplied by the number of units in the chain, while each of the bond vectors from eq 8 was defined by the coordinates of the R3 and S9 beads (i.e., 3, 9, 12, 18,...; 3 + 9*r*, 9 + 9*s*, where *r*,*s* ∈ [0,49] ∩ ). The fitting of the *C*(*n*) vs *n* data by eq 9 was done in a hand written *python* script.

### 3.3 AA Simulations

The following systems were considered in the AA simulations: (1) β-d-GlcNAc monosaccharide; (2) β-d-GlcA monosaccharide; (3) dimer of HA; (4) tetramer of HA; (5) octamer of HA; (6) icosamer of HA. CHARMM36^42,43^ FF was used for all AA simulations. The initial structures of the saccharides as well as the GROMACS-readable parameters were generated from the online server www.charmm-gui.org.^44,45^ The simulations were performed with the GROMACS 2016.4 or 2022 packages.^34^ The saccharide molecules were placed in simulation boxes with dimensions depending on the system type and surrounded by a number of explicit water molecules approximately equal to the system density of 1 g/cm^3^. The number of Na^+^ and Cl^–^ ions was added to each system, taking into account its neutral charge and, for HA-containing systems, also the desired ionic strength value of 0.15 M. The MD simulations were performed under periodic boundary conditions and in the isothermal–isobaric ensemble. The temperature was kept close to its reference value (298 K) using the V-rescale thermostat,^35^ while for the constant pressure (1 bar, isotropic coordinate scaling) the Parrinello–Rahman barostat^36^ with a relaxation time of 0.4 ps was used. The equations of motion were integrated with a time step of 2 fs using the leapfrog scheme.^37^ The translational center-of-mass motion was removed at each time step separately for the solute and the solvent. The TIP3P model of explicit water^46^ was applied and the full rigidity of the water molecules was enforced using the SETTLE procedure.^47^ The bond lengths of the hydrogen containing solute were constrained using the LINCS procedure with a relative geometric tolerance of 10^–4^.^48^ Electrostatic interactions were modeled using the particle mesh Ewald method^49^ with a cutoff of 1.2 nm, while van der Waals interactions (LJ potentials) were switched off between 1.0 and 1.2 nm. Production simulations were run for 100 ns and data were saved to the trajectory every 2 ps.

## 4 Model

### 4.1 Model Parameters

#### 4.1.1 Nonbonded Interactions

When selecting the optimal LJ parameters for the HA disaccharide building block, we considered the chemical character of the mapped functional groups, the AA-derived solvent-accessible surface area (SASA) values, the theoretically predicted log *P* values measured for GlcNAc, and the existing Martini 3 parameters for other biomolecules. For the latter, we considered the chemical similarity of functional groups in saccharides to the parametrized groups that are part of amino acids. The types of beads were chosen from a set of possibilities included in Martini 3 to reproduce thermodynamic data, in particular the free energies of transfer between water and various organic solvents.

Thermodynamic integration (TI) simulations, analyzed using the BAR method, were used to validate the results of the bead type assignment for one of the building blocks of HA, i.e., GlcNAc, because it does not carry an electric charge, unlike the second building block, GlcA. The results of the TI simulations of the GlcNAc monosaccharide allowed to determine a change in the free energy of the system during the transition of the GlcNAc molecule from water to octanol, giving a value of log *P* equal to −3.25, which is close to −3.4 reported by the *Pubchem* database, but slightly lower than the values reported by the *drugbank.ca* database (−2.1) and estimated by the KOWWIN algorithm (Estimation Programs Interface Suite for Microsoft Windows) (−2.25). The large negative value of log *P* is desirable in the context of longer HA chains, which tend to aggregate (see further details).

**Figure 2:**
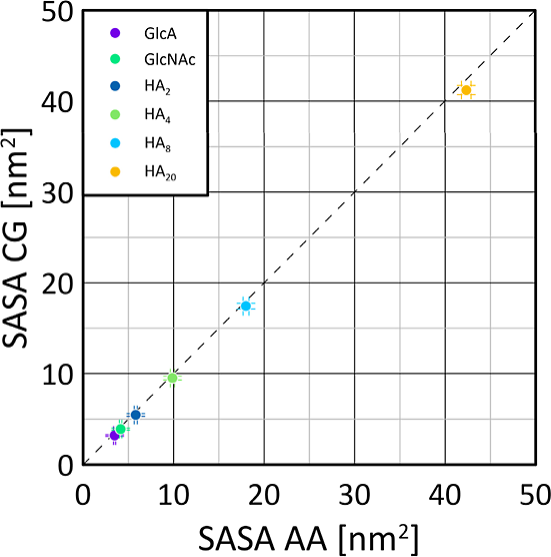
Comparison of the SASA values obtained from either the AA or CG MD simulations. In the legend, the suffix next to HA indicates the number of monosaccharides in a given HA oligo- or polysaccharide.

**Figure 3:**
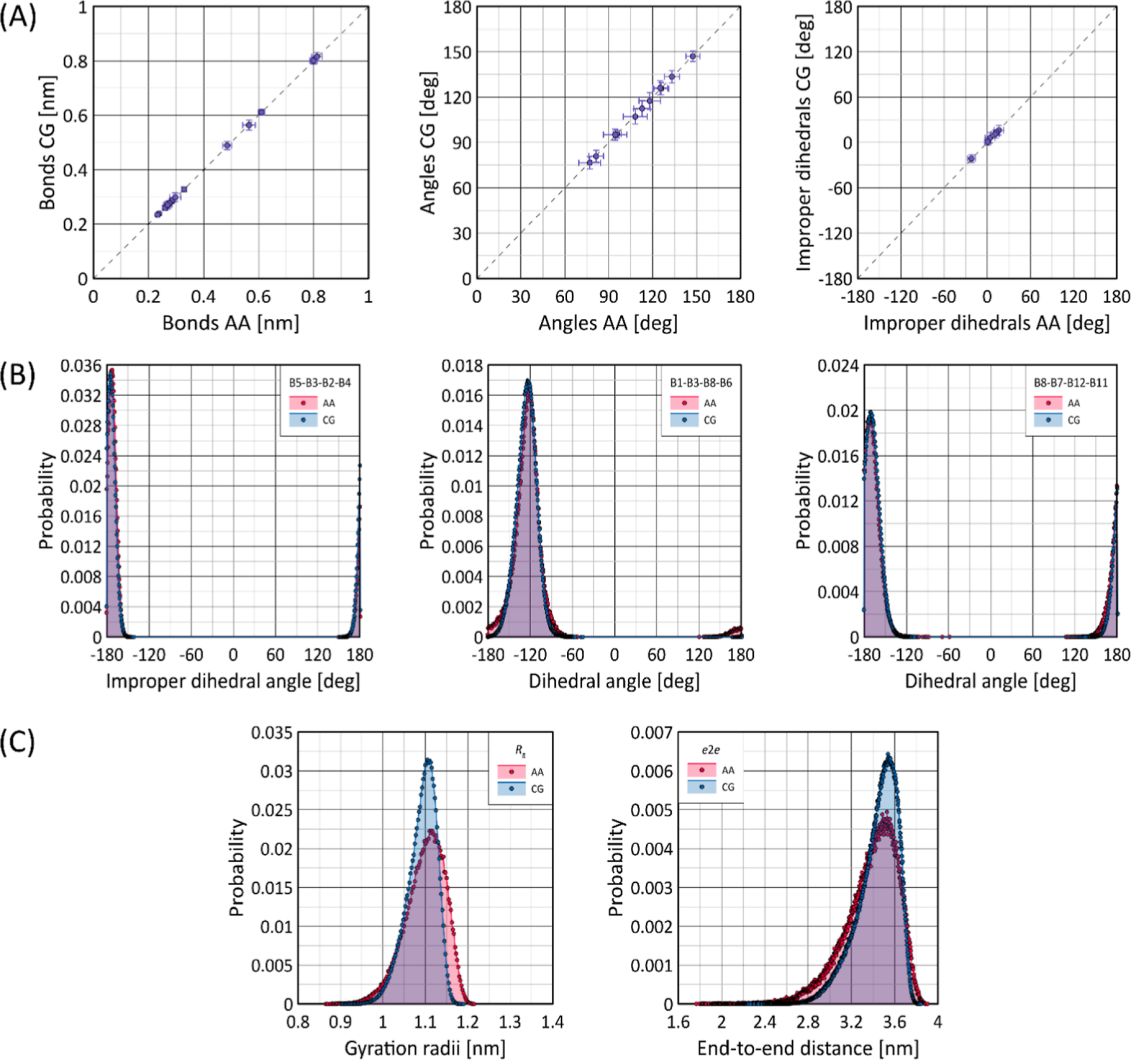
(A) Comparison of the mean values of selected bead–bead distances, bead–bead–bead angles, and improper dihedral angles calculated from unbiased MD simulations within the AA or CG FF for HA. Horizontal and vertical bars indicate the variation associated with a given descriptor, expressed as the standard deviation of the data set. (B) Distributions of selected dihedral angle values calculated within the AA or CG FF for HA. (C) Distributions of radius of gyration (*R*_g_) and end-to-end distance (*e*2*e*) values calculated within the AA or CG FF. Simulations were performed for HA octamers.

The parameters for the whole HA chain include those validated for the GlcNAc monosaccharide and take into account the following properties: (1) the more polar nature of the beads representing the hydroxymethyl, ethanolamine and diol groups (bead 1—**T1***P3r*, bead 3—**R3***P2r* and bead 8—**R8***P3r*, respectively; see Figure 1); (2) the less polar character of the beads representing the central part of the polysaccharide backbone or the acetyl group of GlcNAc (bead 2—**T2***N4r*, bead 4—**S4***N4r*, bead 5—**S5***N5ar*, bead 7—**T7***N4r* and bead 9—**S9***N4r*; see Figure 1); (3) and the polar character and electric negative charge of bead 6 containing the carboxyl group—**S6***Q5n* (see Figure 1). The label *r* was added to the bead types to reduce the self-aggregation properties of the HA chains.

The Martini 3 FF is based on the use of specific pair interactions for LJ parameters instead of the combination rules, as is typical for atomistic FFs. Therefore, we do not provide bead-type-specific LJ parameters but only the bead type, which unambiguously defines the nonbonded interactions for each combination of bead types. The details of how the bead type determines the interactions and the related parameter values are given in Tables S1–S10 in ref (23). Table 2 contains the list of the CG beads used in the final model with the corresponding group in the atomistic representation.

**Table 2:**
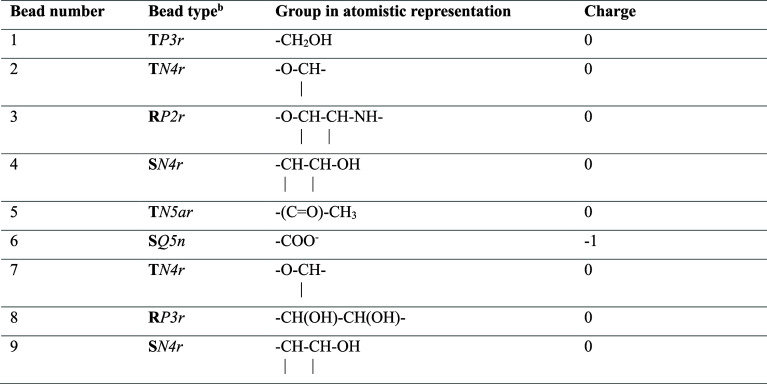
Non-bonded Parameters for All CG Beads Used in the Currently Proposed FF^a^

a Bead numbering in accordance with Figure 1.

b Selected from Martini 3 bead types.

#### 4.1.2 Bonded Interactions

Tables 3 and 4 present the optimized bonded parameters proposed for the GlcNAc and GlcA monosaccharides, respectively. To describe the glycosidic linkages between the GlcNAc and GlcA residues, the parameters for the HA tetramer are presented in Table 5. For the HA dimer (where the nonreducing end is GlcNAc and the reducing end is GlcA), only the parameters for beads 1–9 in Table 5 are used. The repeated, redundant parameters for beads 10–18 (due to the periodicity of the building blocks) are shown in italics. Parameters for oligo- or polysaccharide chains of HA can be obtained from the data in Table 5 by following the rules of periodicity of the HA chain. In particular, to obtain the parameters for the *n*-th dimer within a chain, the parameters for the beads with numbers *Bn* should be used, where *B* is the bead number assigned to the initial HA dimer (*B* = 1–9). The *python 3* program *carbo2martini3_2.0.py* (included in the Supporting Information) allows for the automated generation of oligo- and polysaccharides of arbitrary length as well as the primitive starting structures.

**Table 3:** Parameters for the GlcNAc Monosaccharide (Bond Stretching, Bond Angle Bending and Improper Dihedral Torsion) in the Currently Proposed FF^a^

type	topological pattern	parameters	
bonds		*k*_*b*_ [kJ mol^–1^ nm^–2^]	*b*_0_ [nm]
	B1–B4	10,700	0.270
	B2–B3	19,000	0.284
	B2–B4	25,000	0.354
	B3–B4	19,000	0.288
	B3–B5	9500	0.327
	B4–B5	10,500	0.631
angles		*k*_θ_ [kJ mol^–1^]	θ_0_ [deg]
	B1–B4–B2^b^	230	81
	B1–B4–B3^b^	220	134
	B5–B3–B2^b^	200	101
improper dihedrals		*k*_ξ_ [kJ mol^–1^ deg^–2^]	ξ_0_ [deg]
	B4–B3–B2–B1	170	7.5
	B3–B4–B2–B5	230	–3.5

a Force constants correspond to eqs 1–4.

b Equation 2.

**Table 4:** Parameters for the GlcA Monosaccharide (Bond Stretching, Bond Angle Bending and Improper Dihedral Torsion) in the Currently Proposed FF^a^

type	topological pattern	parameters	
bonds		*k*_*b*_ [kJ mol^–1^ nm^–2^]	*b*_0_ [nm]
	S6–S9	14,000	0.250
	T7–R8	18,000	0.295
	T7–S9	24,000	0.357
	R8–S9	18,000	0.296
angles		*k*_θ_ [kJ mol^–1^]	θ_0_ [deg]
	S6–S9–T7^b^	420	86
	S6–S9–R8^b^	250	137
improper dihedrals		*k*_ξ_ [kJ mol^–1^ deg^–2^]	ξ_0_ [deg]
	S9–R8–T7–S6	300	10.5

a Force constants correspond to eqs 1–4.

b Equation 2.

**Table 5:** Parameters for HA Di- (Only Parameters for B1-9 are Used; Parameters in Italics Result from Periodicity of Disaccharide Building Blocks), Tetra-, Oligo-, and Polysaccharides (Bond Stretching, Bond Angle Bending, and Improper and Regular Dihedral Torsion) in the Currently Proposed FF^a^

type	topological pattern	parameters		
bonds		*k*_*b*_ [kJ mol^–1^ nm^–2^]	*b*_0_ [nm]	
	B1–B4	11,700	0.274	
	*B10–B13*	*11,700*	*0.274*	
	B6–B9	65,000	0.233	
	*B15–B18*	*65,000*	*0.233*	
	B2–B3	73,000	0.232	
	*B11–B12*	*73,000*	*0.232*	
	B7–B8	26,000	0.261	
	*B16–B17*	*26,000*	*0.261*	
	B2–B4	36,000	0.289	
	*B11–B13*	*36,000*	*0.289*	
	B7–B9	50,000	0.257	
	*B16–B18*	*50,000*	*0.257*	
	B3–B4	42,000	0.284	
	*B12–B13*	*42,000*	*0.284*	
	B8–B9	28,000	0.278	
	*B17–B18*	*28,000*	*0.278*	
	B3–B5	38,000	0.330	
	*B12–B14*	*38,000*	*0.330*	
	B2–B9	20,000	0.255	
	*B11–B18*	*20,000*	*0.255*	
	B4–B5	3000	0.615	
	*B13–B14*	*3000*	*0.615*	
	B4–B7	10,000	0.799	
	*B13–B16*	*10,000*	*0.799*	
	B7–B12	400	0.292	
	B7–B11	5000	0.473	
	B9–B13	4000	0.573	
	B2–B12	5000	0.802	
				
angles		*k*_θ_ [kJ mol^–1^]	θ_0_ [deg]	
	B1–B4–B2^b^	345	78	
	*B10–B13–B11*^b^	*345*	*78*	
	B6–B9–B7^b^	630	93	
	*B15–B18–B16*^b^	*630*	*93*	
	B1–B4–B3^b^	270	126	
	*B10–B13–B12*^b^	*270*	*126*	
	B6–B9–B8^c^	345	146	
	*B15–B18–B17*^c^	*345*	*146*	
	B5–B3–B2^b^	500	126	
	*B14–B12–B11*^b^	*500*	*126*	
	B3–B2–B9^b^	150	93	
	*B12–B11–B18*^b^	*150*	*93*	
	B2–B9–B8^b^	150	116	
	*B11–B18–B17*^b^	*150*	*116*	
	B2–B9–B6^b^	150	80	
	*B11–B18–B15*^b^	*150*	*80*	
	B8–B7–B12^b^	250	110	
	B7–B12–B11^b^	200	120	
	B7–B12–B13^b^	150	66	
improper dihedrals		*k*_ξ_ [kJ mol^–1^ deg^–2^]	ξ_0_ [deg]	
	B4–B3–B2–B1	150	16	
	*B13–B12–B11–B10*	*150*	*16*	
	B5–B3–B2–B4	170	–173	
	*B14–B12–B11–B13*	*170*	*–173*	
	B9–B8–B7–B6	350	13	
	*B18–B17–B16–B15*	*350*	*13*	
	B2–B3–B9–B4	170	5	
	*B11–B12–B18–B13*	*170*	*5*	
	B9–B2–B8–B7	300	13	
	*B18–B11–B17–B16*	*300*	*13*	
	B7–B8–B9–B12	500	1	
	B12–B11–B13–B7	400	–18	
regular dihedrals		*k*_φ_ [kJ mol^–1^]	φ_0_ [deg]	*m*
	B1–B3–B8–B6	–32	–138	1
	*B10–B12–B17–B15*	*–32*	*–138*	*1*
	B8–B7–B12–B11	–60	–165	1

a Force constants correspond to eqs 1–5. The bead numbering refers to the first four residues in the HA polysaccharide chain, starting from the nonreducing end (see Figure 1). Parameters for the longer HA chains can be obtained by following the rules of periodicity of the HA chain. In particular, the parameters of the beads *Bn* should be used to obtain the parameters for the *n*-th dimer within a chain, where *B* is the bead number assigned to the initial HA dimer (*B* = 1–9).

b Equation 2.

c Equation 3.

As mentioned in our previous work,^27^ such a parametrization strategy allows to use the parameters of monosaccharides to build CG models for more complex saccharides. Thus, the parameters for the HA tetra-, oligo-, and polysaccharides (Table 5) are similar to those for the GlcNAc and GlcA monosaccharides (Tables 3 and 4), with some modifications and the addition of components responsible for modeling glycosidic linkages.

Summarizing, the parameters from Tables 3 and 4 (and their counterparts from Table 5) describe the conformation of monosaccharide fragments of HA chains, including ring flexibility (restricted to the ^4^*C*_1_ conformation, characteristic of all glucopyranose residues) and rotation of exocyclic substituents. In addition, Table 5 contains parameters modeling the flexibility of glycosidic linkages, where the conformation of both 1 → 3 and 1 → 4 linkages is restricted to the exploration of a main free energy minimum (corresponding to the exo-syn conformation). The maintenance of the correct conformation of the glycosidic linkages is primarily controlled by potentials related to the values of the torsion angles B1–B3–B8–B6 (1 → 4 linkage) and B8–B7–B12–B11 (1 → 3 linkage), but also by potentials related to the improper dihedral values for the CG beads adjacent to the B2–B9 and B7–B12 linkages (notation according to Figure 1). Conformational changes in the glycosidic linkages associated with the migration of the conformation to the anti-ϕ and anti-ψ geometries cannot be modeled by the single-well, harmonic potentials used to define the improper dihedrals (eq 4).

### 4.2 General Remarks

The parameters collected in Tables 3–5 have been developed, tested and validated under the assumption that the first covalently bonded neighbors are excluded from any nonbonded interactions. It should be noted that in some cases bonds exist between nonadjacent beads. They have been introduced to control the geometry of triplets of beads, where the geometry of such triplets becomes almost linear, leading to potential numerical errors and instability of the simulation. For the same reasons, the regular bond angle term (eq 2) was replaced by a restricted angle term (eq 3) for flexible bond angles with optimal values close to 180°.

In addition, specific parameters were added to ensure the stability of the glycosidic linkage between GlcA and GlcNAc residues, as some of these linkages are prone to linearization. These additional parameters help to maintain the correct geometric configuration of the linkage during simulations. Overall, the parameters developed for HA-containing systems allow for stable MD simulations at a time step of 0.01 ps (Table 5), while systems containing monosaccharides (Tables 3 and 4) remain stable at a time step of 0.03 ps.

## 5 Properties of the Investigated Systems

The following subsections present the results obtained using the newly developed set of parameters to study various HA-containing systems. These results are intended to validate the accuracy of the FF, some were used to refine the final parameters for HA, and some present biologically relevant results for HA-containing systems.

### 5.1 SASA

Solvent-accessible surface area (SASA) parameter values were calculated at both CG and AA levels of resolution for GlcNAc and GlcA monosaccharides, HA dimers, tetramers, octamers, and icosamers. The results are shown in Figure 2 and demonstrate that the CG models accurately reproduce the molecular surface and volume properties essential for Martini 3-based models,^23,28^ with remarkably small relative and absolute deviations from atomistic data and natural fluctuations of the SASA parameter of comparable magnitude for both CG and AA data.

### 5.2 Conformational Properties

The parameters collected in Tables 3–5 were used to develop a set of models for GlcNAc and GlcA monosaccharides and HA octamers, which were then used to validate the conformational properties against predictions from AA MD simulations. This validation was performed by comparing the distribution of selected conformational descriptors: (1) distances between specific CG bead pairs; (2) angles between sets of three beads; (3) improper dihedrals defined by groups of four beads; (4) regular torsion angles defined by quadruplets of beads; (5) radii of gyration; (6) end-to-end distances. Given the large number of possible bead combinations to define each descriptor, the analysis was focused primarily on those directly related to specific FF terms (e.g., bead–bead bonds or dihedral angles defined on rotatable bonds). For HA octamers, the analysis for descriptors (1–4) always concerned the central residue(s) of the chain. Covalent bead–bead bonds, bond angles, and improper dihedral terms were modeled using harmonic potentials (see Sections 2 and 4). To facilitate comparisons for these descriptors, only averages and their fluctuations (standard deviations) are reported. For illustrative purposes, a selected improper dihedral angle with average value close to 180° and the two regular dihedral angles are presented as distributions obtained from both AA and CG simulations. The results are shown in Figure 3. In addition to these analyses, a comprehensive comparison of the distributions of many other conformational descriptors was performed at the parametrization and data analysis stages.

In most cases the agreement between the AA and CG data is excellent or at least satisfactory. For bonds, bond angles, and improper dihedrals, the most significant deviations arise from the inherent asymmetry of the AA distributions, which are approximated by symmetric parabolic potentials in the CG model. However, such deviations are rare, and the asymmetry and deviation from unimodal character in the distributions are usually minimal, resulting in a reasonable level of agreement between the AA and CG data (see Figure 3). The dihedral angle distributions shown also agree well with the corresponding AA distributions. In general, the CG model accurately captures the conformational characteristics of the bonded parameters of HA, confirming the reliability of the final CG model. Finally, it is worth noting the good agreement between the large-scale polymer properties (radius of gyration and end-to-end distance) calculated at the CG and AA levels. The mean values and even the nonsymmetric character of the AA-derived distributions are reasonably well reflected by the corresponding CG model (Figure 3).

Although we have not directly examined the agreement between the predictions of our CG model and existing crystal structures and NMR data of HA chains, it is worth noting that the atomistic CHARMM force field on which the reference data were based accurately reproduces these structural data, for example in the context of the preferred conformation of glycosidic bonds (see Figure 4 in ref (50)) or predictions of the 3D structure of the hyaluronan chain using the local conformation of disaccharide building blocks.^17^

### 5.3 Aggregation Properties

The aggregation behavior of the HA models was investigated by MD simulations of the systems with different salt concentrations (CaCl_2_ or NaCl) and 25 HA octamers. The investigation also included MD simulations with different amounts of 100-mers of HA (4, 8, 12, 24) in the presence of physiological concentration of NaCl. The results showed that HA octamers tended to aggregate under the conditions studied, probably due to neutralization of negatively charged carboxyl groups (S6 beads) by Na^+^ or Ca^2+^ cations. This neutralization caused the HA chains to behave as neutral molecules. It was also observed that the aggregation properties were less pronounced with Ca^2+^ cations compared to Na^+^ cations. This can be attributed to several factors: Ca^2+^ ions are surrounded by more water molecules due to their larger hydration radius, which may hinder their ability to effectively neutralize carboxyl groups on HA molecules. Additionally, after binding, Ca^2+^ interacts more strongly with water molecules, stabilizing HA chains in solution and reducing their tendency to aggregate. Moreover, Ca^2+^ ions can interact with two carboxyl groups within the same HA molecule, leading to intramolecular interactions that alter the conformation of the molecule and potentially reduce its ability to participate in intermolecular aggregation. Finally, Ca^2+^ can form intermolecular “bridges” between two HA molecules, changing their conformation and potentially affecting their ability to form intermolecular aggregates.

**Figure 4:**
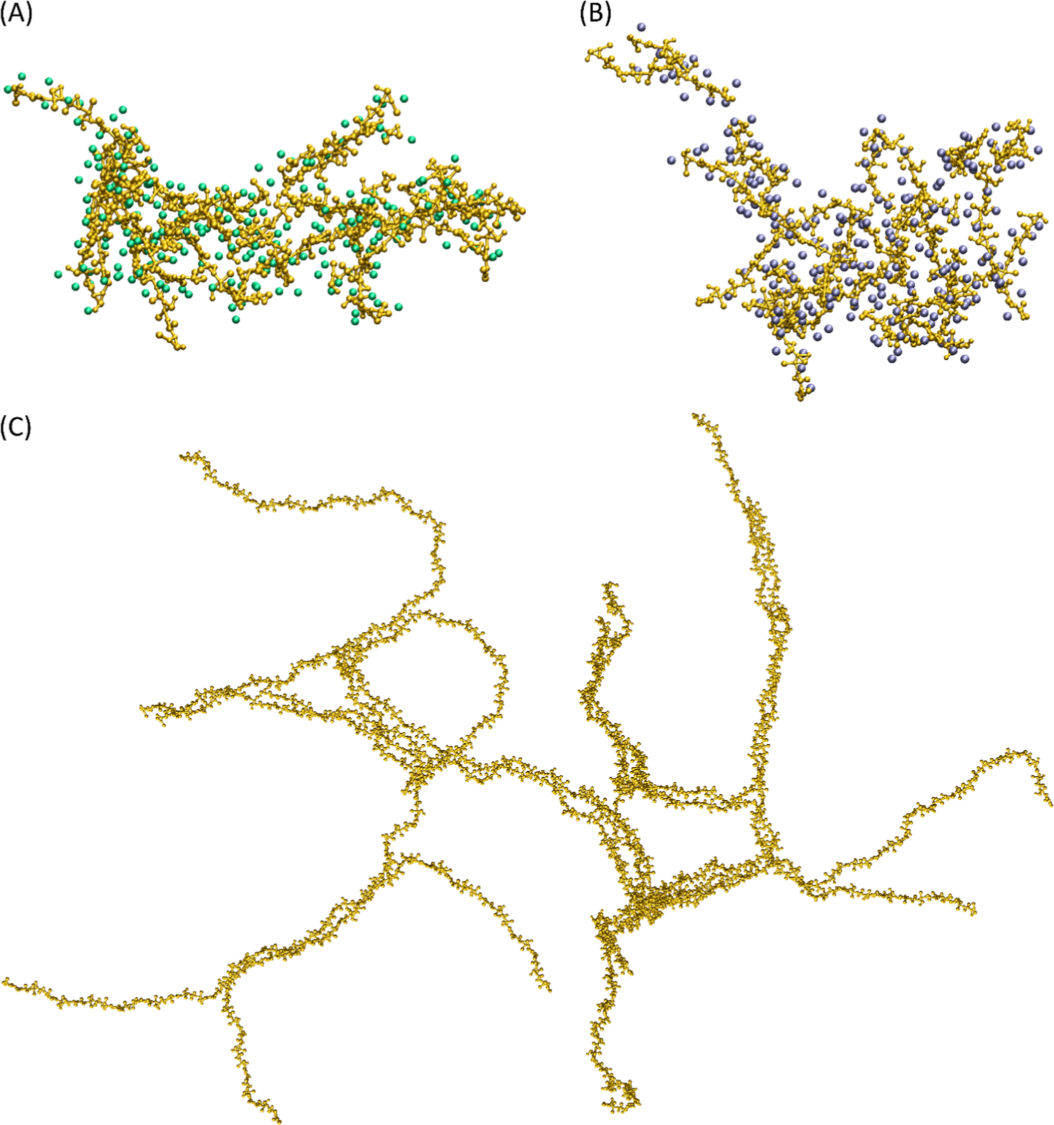
Visualization of HA aggregates obtained in CG MD simulations. HA chains are shown in yellow. (A) The aggregate of 25 HA octamers in the presence of Na^+^ cations (shown as green spheres). (B) The aggregate of 25 HA octamers in the presence of Ca^2+^ cations (shown as blue spheres). (C) The aggregate of 12 HA polymer chains of length of 100 mers.

The simulation results are in qualitative agreement with the experimental data^51^ and show that HA chains maintain their extended conformation regardless of the concentration of ions in solution while forming aggregates, which is important for their biological functions, such as forming the extracellular matrix, imparting viscoelastic properties to tissues, and regulating cell behavior. Exemplary aggregates of 25 HA octamers in the presence of Na^+^ and Ca^2+^ cations and an aggregate of 12 HA polymer chains of length of 100 mers are shown in Figure 4.

### 5.4 Persistence Length

**Figure 5:**
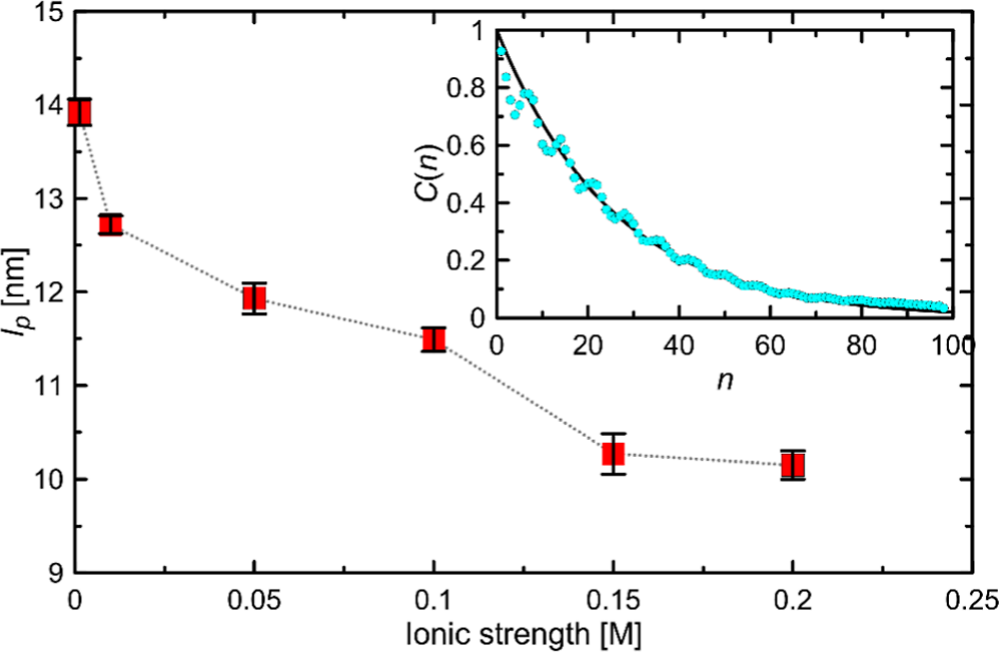
Dependence of the persistence length value determined in CG MD simulations for the HA chain of length of 100 mers on the ionic strength of the solution (expressed as NaCl concentration). The inset plot shows a sample autocorrelation function *C*(*n*) determined for one of the systems and fitted by eq 9.

The persistence length calculations involved simulations of systems containing a single 100-mer of HA at various concentrations of NaCl, as detailed in Table 1. These simulations were performed to evaluate how different ionic strengths affect the structural properties of HA, with particular emphasis on its stiffness and overall conformational behavior in solution, and to compare with the results of experimental studies. The experimentally determined persistence length values for HA vary over a wide range depending on the conditions under which the measurements were made and the method used to determine the persistence length. For example, in ref (52), the persistence length values (total persistence length) range from 8.7 to 18.4 nm, depending on the ionic strength, with the so-called intrinsic persistence length being about 9 nm. Reference (53) reports an even smaller value, about 4 nm, with measurements corresponding to low pH (which in practice forces HA protonation) and high ionic strength. Similarly low values are obtained in systems with high ion concentrations, where the effect depends on the type of metal cation (Na^+^ or Ca^2+^) due to cation binding to HA.^54^ Regardless, all studies consistently predict a decrease in persistence length with increasing ionic strength. The same trend exists in the data generated using the CG model proposed in this work (see Figure 5). In particular, the experimental values of total persistence length for the conditions closest to the physiological ionic strength of ref (52) (i.e., 8.7 and 12.8 nm) are close to the value obtained from modeling, around 10.2 nm (for an ionic strength of 0.15 M). The drastic decrease in persistence length with increasing ion concentration cannot be observed because of: (1) inaccuracies in the CG Martini 3 model in describing metal cation-organic anion binding; (2) limitations in modeling the rotation of the glycosidic linkage associated with conformational transitions from syn-exo rotamers (the major conformation) to anti-ones. The latter factor may contribute to chain stiffening in systems where the equilibrium of glycosidic linkage conformations is significantly shifted toward the anti-conformers.

### 5.5 Interactions with the Lipid Bilayer

The membrane model was constructed using the phospholipids POPC, POPE, POPS and cholesterol, with all FF parameters assigned according to Martini 3.^23,24,55^ The lipid composition was based on the mammalian membrane composition described in another study,^56^ except for palmitoylsphingomyelin, as the parameters for this molecule have not yet been developed for Martini 3. Three systems were simulated: a reference system with only the lipid membrane, a second system with the membrane and 20 HA dimers, and a third system with the membrane and a single HA octamer.

**Figure 6:**
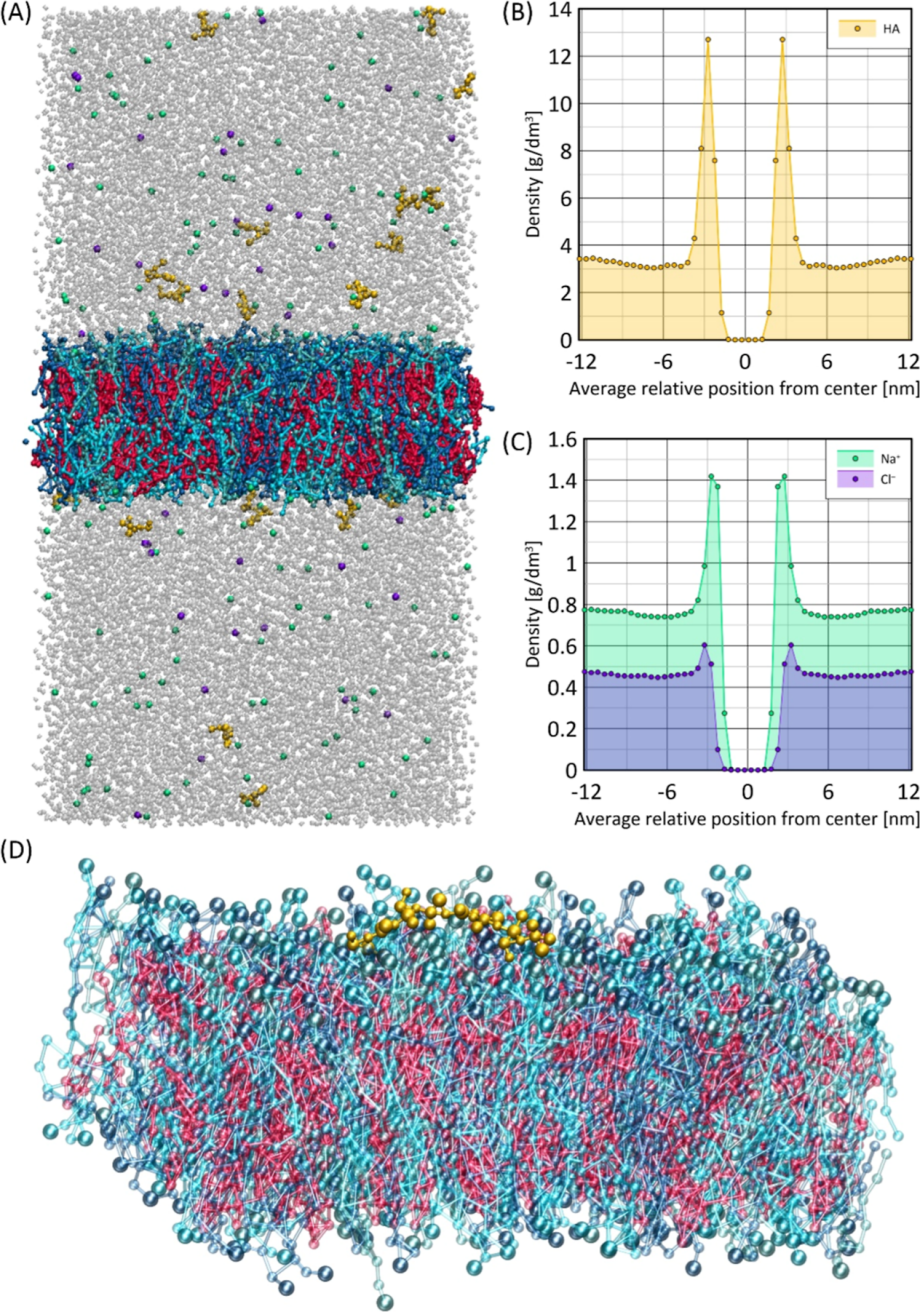
(A) Snapshot of the simulation box containing the lipid bilayer. Phospholipids are shown in various shades of blue, cholesterol in red, HA dimers in yellow, Na^+^ ions in green, Cl^–^ ions in purple, and water molecules in gray. (B) Plot of the density profile of HA as a function of the average relative position from the center of the simulation box. (C) Plot of the density profiles of Na^+^ and Cl^–^ ions as a function of the average relative position from the center of the simulation box. (D) Snapshot of the lipid bilayer interacting with a single HA octamer.

Figure 6 shows the graphical representation of the lipid bilayer with 20 HA dimers and Na^+^ and Cl^–^ ions, the plots showing the density profiles of HA and Na^+^ and Cl^–^ ions relative to the average position from the center of the box, and the representation of the lipid bilayer with a single HA octamer. The results of the simulations indicate that Na^+^ ions interact more intensely with the membrane surface than Cl^–^ ions. This behavior can be explained by the electrostatic interaction between the positively charged sodium ions and the negatively charged lipid head groups, especially POPS, which carries a net negative charge (in contrast, POPC and POPE are zwitterionic, with both positive and negative charges in their head groups, but are overall neutral).

Interestingly, the interaction between HA dimers and the membrane surface was found to be an order of magnitude stronger than that of Na^+^ ions, in agreement with other studies.^57,58^ This can be attributed to the interaction of Na^+^ ions with the carboxyl groups of HA, which neutralizes the HA charge and facilitates its interaction with the zwitterionic lipid head groups. Membrane surface-HA interaction can consequently lead to the adsorption of HA oligomeric chains on a membrane surface, as shown in Figure 6. However, in the case of longer polymeric chains, analogous adsorption may not be as evident.^57,58^ HA and lipid beads interaction pattern displays an nonuniform character, with the *P3r* beads of HA interacting more strongly with the beads of the -PO_4_ group of the lipid head, while the *N4r* beads interact more strongly with the beads of the –NR_3_^+^ group in the lipid head. This interaction results in an increased presence of HA near the membrane surface, potentially facilitating its entry into binding sites on membrane-associated proteins. In the system containing a single HA octamer, the octamer exhibited dynamic, fluctuating interactions with the membrane, sometimes moving closer to the surface and sometimes drifting away, indicating a variable interaction pattern and reversible adsorption process.

### 5.6 Interactions with Proteins

#### 5.6.1 CD44 Protein and Its Interactions with HA Octasaccharide

The CD44 protein is a transmembrane protein that plays a key role in cell adhesion, migration and other cellular processes, including the development of cancer. It consists of several functional parts: the extracellular domain, which includes the hyaluronan-binding domain, the transmembrane segment, which anchors the protein to the cell membrane, and the cytoplasmic domain, which is involved in intracellular signaling pathways. The extracellular part of CD44 interacts with HA, which induces various biological processes.

The availability of the crystallographic structure of the murine CD44 protein bound to HA (PDB: 2JCR)^59^ made it possible to investigate which amino acid residues are important for binding to HA. It was found that one of the key amino acids in the murine CD44 protein responsible for binding to HA is Arg45 (corresponding to Arg41 in the human protein), and two basic protein conformations, A and B, were described that differ in the orientation of the Arg45 side chain and its ability to form a tight contact with HA. This crystallographic structure provided an opportunity for further simulation-oriented studies of the interaction of CD44 with HA. For example, the following issues were investigated: the dynamic properties of the interaction between CD44 and HA,^60,61^ the role of Tyr161 in a possible partial unfolding of CD44,^62^ and the possibility of noncrystallographic binding sites of HA interacting with CD44.^63^ There are a number of more or less subtle differences between the results obtained using different computational setups, including different force fields.^64^ The results reported in ref (63) are particularly important in the context of the present study. Namely, the three main binding sites in the interaction of CD44 with HA have been identified: crystallographic, parallel and upright.^63^ They correspond to three different binding patterns that can exist simultaneously. The crystallographic-like binding position has been identified as the strongest, but not the most accessible in the early stages of binding. The other two binding positions, parallel and upright, are metastable and frequently found in simulations, suggesting that they play an important role in the early stages of binding.

**Figure 7:**
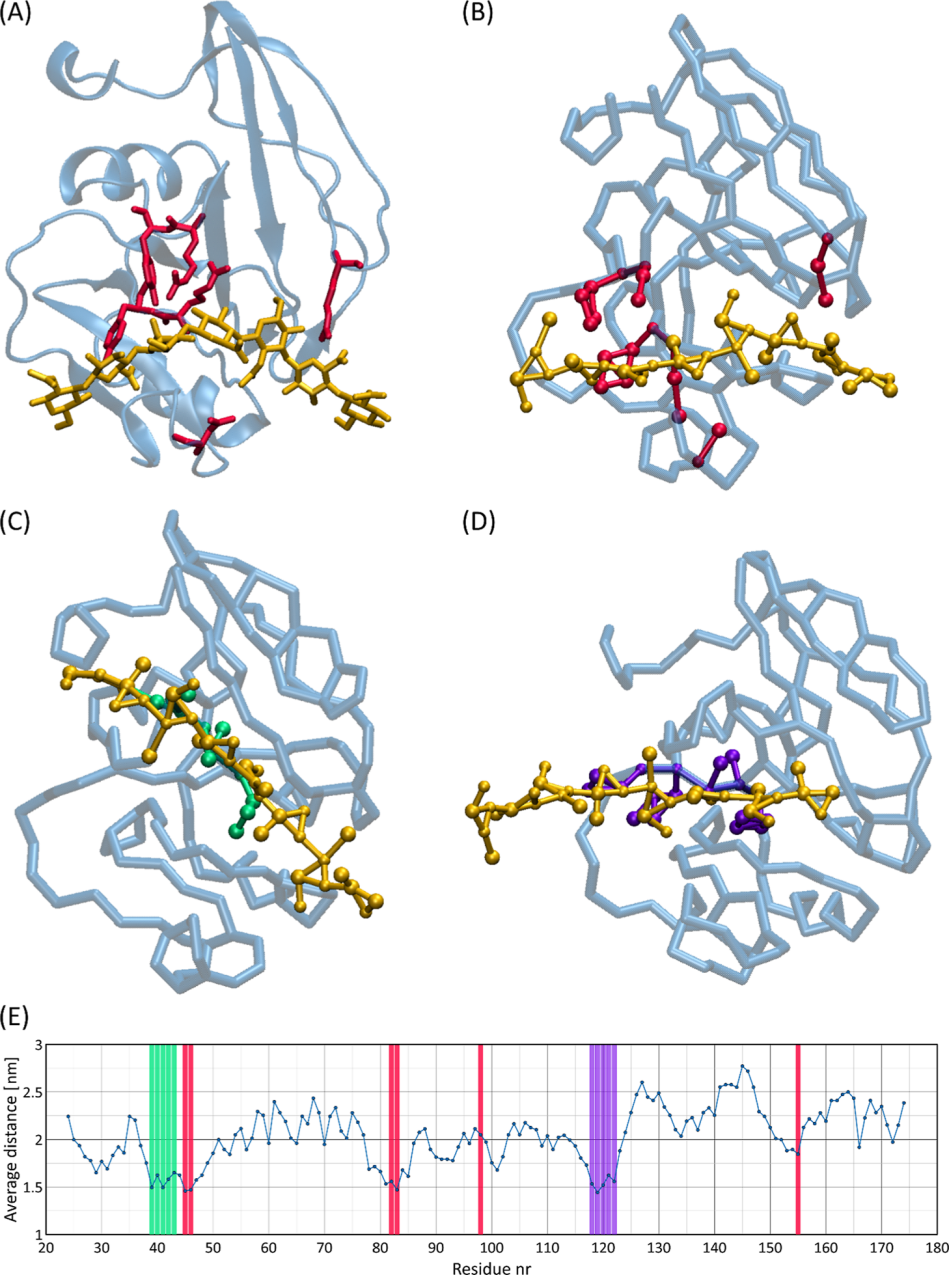
Analysis of the HA binding sites of the CD44 protein: (A) the AA crystallographic structure of the CD44 protein in complex with an HA octasaccharide (PDB: 2JCR). (B) The CG structure reflecting the crystallographic binding site observed in the AA structure (*red* binding site). (C) The *green* binding site observed in the CG simulations, similar to the binding site identified in ref (65). (D) The *purple* binding site identified in the CG simulations, similar to the “parallel” binding site described in the theoretical study of ref (63). (E) A plot showing the average distance of the HA octasaccharide from different amino acid residues on 2JCR, generated using the *gmx mdmat* tool. The color of the vertical bars corresponds to the group of amino acids highlighted in panels (A–D).

The results of current CG MD simulations of the system containing HA and CD44 protein confirm these observations. Figure 7 shows the data for the studied system containing the HA octamer and the HA binding domain of the CD44 protein. In analogy to the results of ref (63), three major binding sites were identified, tentatively named *red*, *purple* and *green* (as shown in Figure 7). The *red* site corresponds to the crystallographic binding site^59^ where the major binding residues are Arg45 and Tyr46. This binding mode is one of the strongest and most stable among those identified, confirming its critical role in stabilizing the CD44-HA complex. The *purple* binding site identified in our simulations is similar to the “parallel” binding site of ref (63). This binding mode shows frequent contacts with HA in the early stages of binding and the interaction is characterized by HA contacting CD44 on a larger surface area of the protein, but with less specificity, which may indicate that HA can move further into the crystallographic (*red*) binding site. The *green* binding site, consisting of amino acid residues indirectly identified as relevant for HA binding by experimental mutagenesis study,^65^ shows moderate stability and less specific binding than the crystallographic binding site. The binding site alternative to the crystallographic site may be transient in cases where HA binds to the CD44 protein either from aqueous solution or from the cell membrane surface. The present results show that the proposed CG model of HA is able to reproduce both experimental and theoretical data on the CD44-HA interaction. The three binding sites identified confirm the results of previous studies and demonstrate the ability of the HA model to explore the HA binding sites in the CD44 protein and the complex biomolecular interactions involved in this phenomenon.

#### 5.6.2 Hyaluronate Lyase and Its Interactions with HA Tetrasaccharide

Hyaluronate lyase (SpnHL) is a bacterial enzyme that breaks down HA as it binds to body tissues. This destruction allows the bacterium to enter the body more easily. The SpnHL protein consists of two main parts: an α-helical domain at one end and a β-sheet domain at the other, between which the active site of the enzyme is located. Crystallographic studies have shown which amino acids play a key role in HA binding and cleavage.^66^

**Figure 8:**
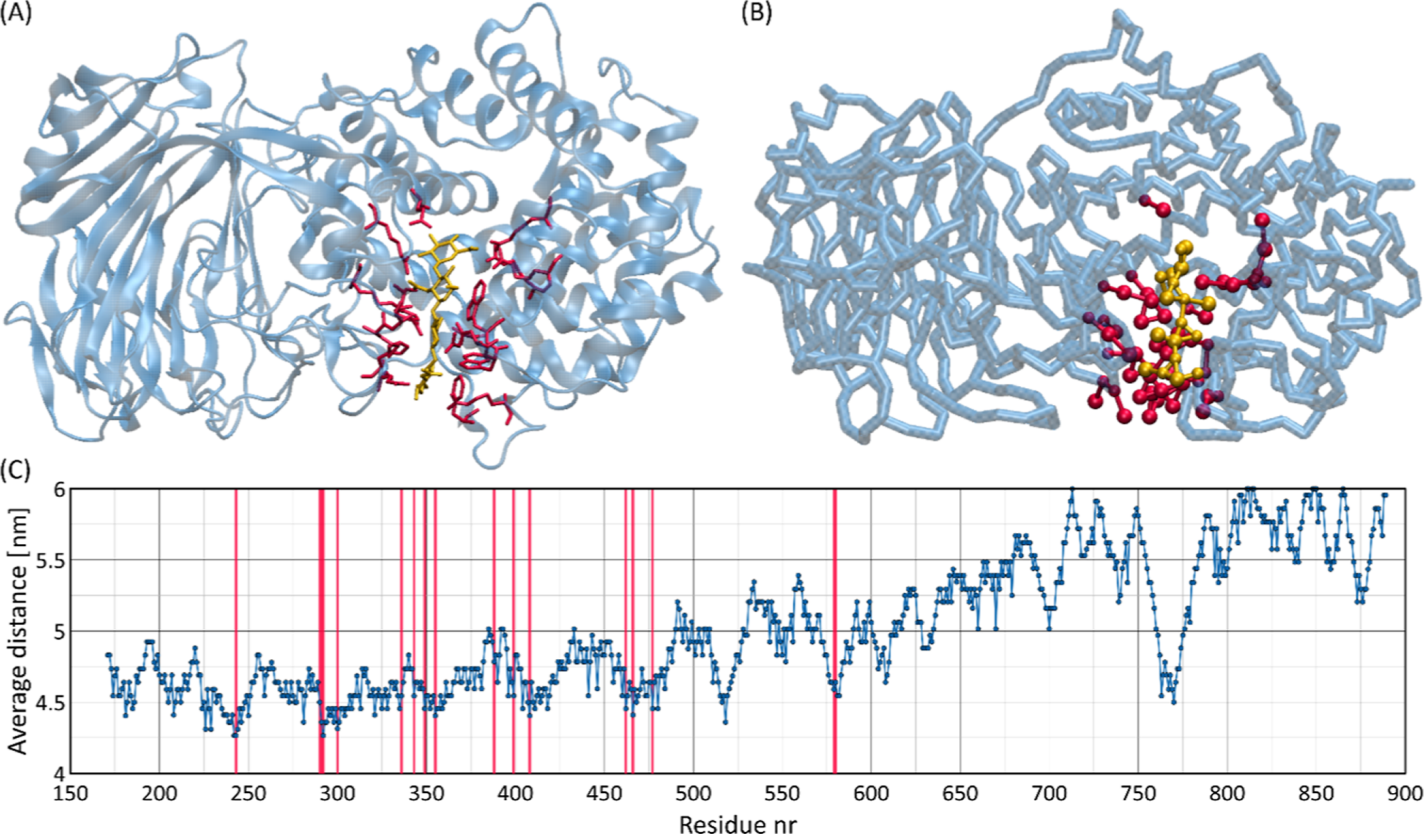
Analysis of the HA binding site of SpnHL protein: (A) the AA crystallographic structure of SpnHL protein in complex with an HA tetrasaccharide (PDB: 1LXK). (B) The CG structure reflecting the crystallographic binding site observed in the AA structure. (C) A plot showing the average distance of the HA tetrasaccharide from different amino acid residues on 1LXK, generated using the *gmx mdmat* tool.

MD simulations within the currently proposed CG model of the HA tetrasaccharide and the SpnHL protein (from *Streptococcus pneumonia*; PDB: 1LXK) provide observations similar to those from crystallographic studies. Figure 8 illustrates the data for one of the systems under consideration. The plot shows the average distance between the HA tetrasaccharide and different amino acid residues. The analysis of the average distances as well as the insight into the MD trajectories confirm that the crystallographic binding site is correctly identified during the MD simulations, although the binding is dynamic and multiple binding and unbinding events can be observed. These results indicate that the proposed CG model of HA is able to identify the binding site in SpnHL; however, the highly dynamic nature of the binding suggests that the properties determined may be qualitative rather than quantitative in nature.

#### 5.6.3 Glucuronoyl Esterase and Its Interactions with GlcA Monosaccharides

The glucuronoyl esterase *Ot*CE15A (PDB: 6SYV) from the bacterium *Opitutus terrae* is an enzyme involved in the cleavage of ester bonds between lignin and GlcA in the plant cell wall. Crystallographic studies revealed the presence of a specific binding site in *Ot*CE15A that interacts with GlcA.^67^

**Figure 9:**
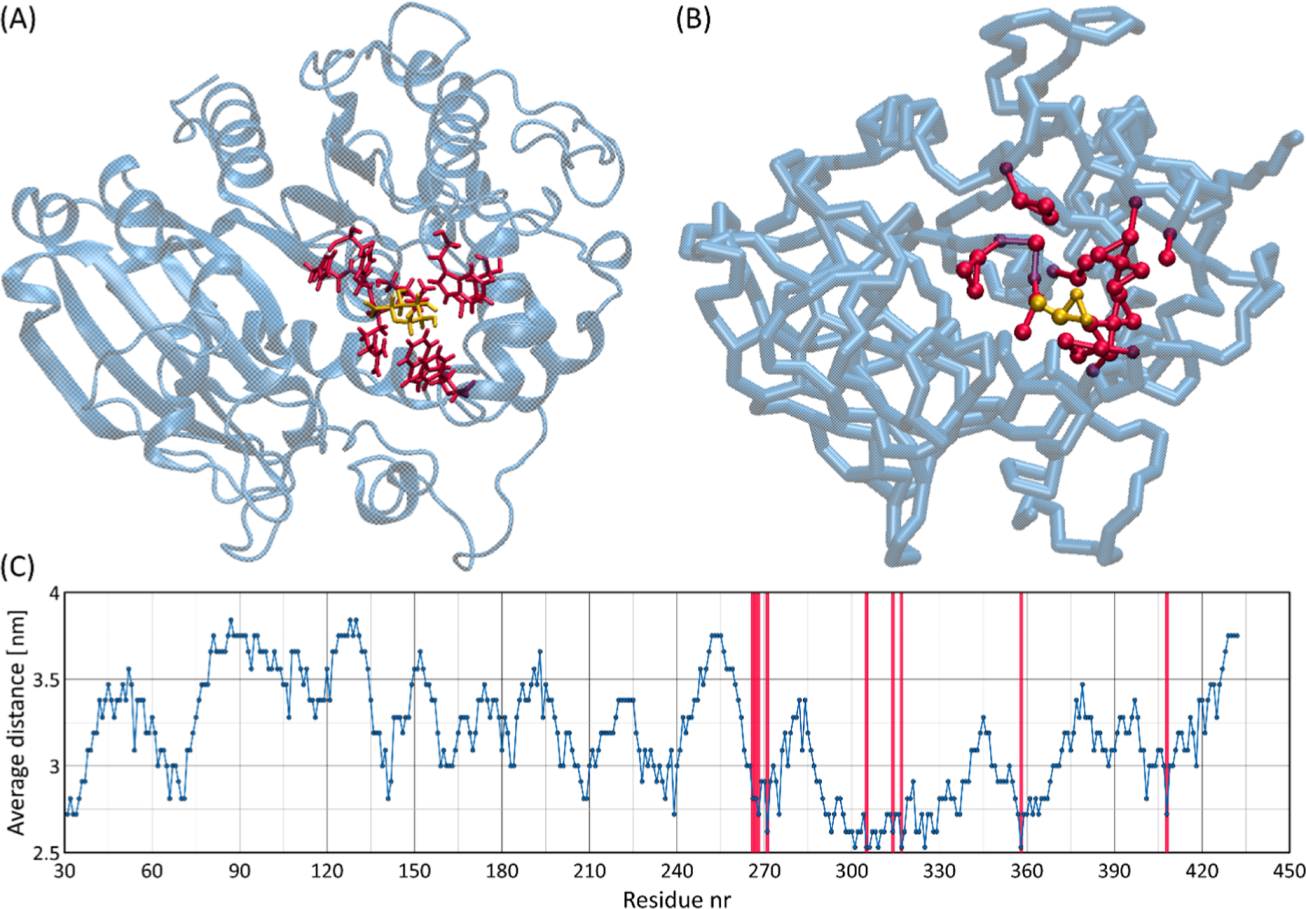
Analysis of the GlcA binding site of *Ot*CE15A protein: (A) the AA crystallographic structure of *Ot*CE15A protein in complex with a GlcA (PDB: 6SYV). (B) The CG structure reflecting the crystallographic binding site observed in the AA structure. (C) A plot showing the average distance of 10 GlcA monosaccharides from different amino acid residues of *Ot*CE15A, generated using the *gmx mdmat* tool.

MD simulations of the interactions between 10 GlcA monosaccharides and the *Ot*CE15A protein, performed within the currently proposed CG model, provide observations similar to the crystallographic data. Figure 9 illustrates the data for one of the systems studied. The results confirm that GlcA interacts with the crystallographically defined binding site of *Ot*CE15A. As shown by the average distance between 10 GlcA monosaccharides and different amino acid residues, GlcA monosaccharides bind to the crystallographic binding site, but as in the previous case of the SpnHL protein, the binding has a dynamic character and multiple binding and unbinding events are observed. Again, the results indicate that the CG model of GlcA is well suited for the identification of the binding site in *Ot*CE15A, but not necessarily for the exact and quantitative determination of the thermodynamics of the binding process.

## 6 Model Applicability, Limitations and Potential Refinements

The described CG model extends the applicability of CG FFs from the Martini 3 family, allowing for highly efficient computational simulations in systems containing HA. The increase in computational efficiency is highly desirable in systems with linear polysaccharides, as it allows a significant extension of the simulated chain lengths. This in turn enables simulations beyond the regime of a linear relationship between chain length and radius of gyration or end-to-end distance,^41^ bringing the simulated HA chains closer to their natural sizes.

The validation scope of the newly developed model includes HA interactions with other biomolecules (proteins and lipid membranes). In addition, the fundamental philosophy of the Martini 3 FF—its carefully designed cross-interaction parameters for different CG bead types—allows our model to be applied to simulations of natural biological systems, which often have a highly heterogeneous composition. Examples include the pericellular hyaluronan coat anchored to the cell membrane via CD44, or macromolecular complexes formed by hyaluronan with proteoglycans in the extracellular matrix.^50^ In this context, an additional advantage of the proposed model is its ability to identify HA-binding sites on protein surfaces without prior knowledge of their locations. Furthermore, the increased accessible simulation time scale (hundreds of microseconds) allows the sampling of multiple alternative HA-protein binding mechanisms.

In the context of studying such complex biological systems, it is crucial to develop compatible CG models for other system components–primarily other glycosaminoglycans (including sulfation patterns) and glycans linked to proteins via glycosylation.

The currently proposed CG model of HA has a number of limitations, most of which are similar to those of the previous model developed for glucopyranose-based carbohydrates^27^ and coarse-grained models in general.

1. *Nonbonded parameters*: Due to the inability to accurately validate the change in free energy during the transition from water to octanol of the entire HA molecule, some other chemically reasonable combinations of bead types may also provide appropriate properties of the nonbonded parameters of the HA model. The main motivation for introducing such potential changes may be to alter the strength of self-interaction within a single HA chain or between different HA chains, as well as between HA and other biomolecules or solvents.
2. *Orientation-dependent interactions*: As in the previous model for glucopyranose carbohydrates,^27^ interactions with a strong orientation dependence (e.g., hydrogen bonding) are modeled by orientation-free LJ interactions between beads. This may lead to an inaccurate representation of HA self-assembly in aqueous solutions or interactions with other biomolecules.
3. *Protein binding*: Although binding sites have been identified in the tested examples of HA and GlcA models with their respective binding proteins, the model may not accurately reflect the kinetics and thermodynamics of carbohydrate binding to proteins; the source of potential inaccuracies may lie in the issues mentioned in points 1 and 2.
4. *Conformational changes*: The model is not able to reproduce the rotation around the glycosidic linkages corresponding to the migration from exo-syn to *anti*-conformations. This can lead to excessive stiffness of the HA chain, especially for longer HA chains, and is a likely reason for the overestimation of the persistence length (see Section 5.4). Furthermore, none of the possible ring distortions are reflected by the model. However, since both residues present in the HA chain have the *gluco* configuration of the ring substituent, the ring is almost always in the regular chair (^4^*C*_1_) conformation, and this shortcoming has a minor effect on the HA behavior.

These limitations can be overcome by introducing alternative parameters, additional constraints, or changing the functional forms of the potentials, which is planned in our future studies (in particular, systematic exploration of the issue of small-scale conformational transitions such as ring distortion and glycosidic linkage rotation within the CG models).

## 7 Conclusions

The CG model for HA, compatible with the new version (3) of Martini FF, has been developed and validated. In addition to the HA polysaccharide, parameters for its constituent monosaccharides, GlcNAc and GlcA, were also developed. The parametrization procedure followed the usual Martini-related methodology, e.g., the nonbonded parameters were chosen to reproduce the water/octanol partition coefficients and the solvent-accessible surface area, and the bonded parameters were adjusted using data from AA simulations concerning the distribution of different conformational descriptors. An extensive series of MD simulations was performed to characterize various aspects of HA behavior within the newly proposed model. For example, the aggregation capabilities of HA chains of different lengths were tested in solutions containing different concentrations of Na^+^ and Ca^2+^ ions. The structural and conformational properties of single-chain HA within the newly developed model were further investigated by calculating the persistence length in solutions of different ion concentrations; the experimentally inferred trend of decreasing persistence length with increasing ionic strength of the solution was correctly reproduced. The model was also tested in the context of HA interactions with a lipid bilayer. In addition, the ability of HA and GlcA to interact with the corresponding proteins that bind them in biological systems was tested. It was found that the proposed model is able to correctly identify the binding sites of proteins without any prior assumptions about the location of the binding sites. This opens up the possibility of using our model in a blind search for HA binding sites in proteins.

The newly developed model can be further refined and extended. In our future work, we plan to investigate: (1) a further extension of the saccharide-dedicated CG models to cover other glycosaminoglycans, and (2) improving the accuracy of the existing models by introducing additional features that will enhance their ability to better reproduce the conformation of long polysaccharide chains in the context of rotation of glycosidic linkages as well as ring distortions.

## Supporting Information Available

*python 3* script (*carbo2martini3_2.0.py*) for generating primitive starting geometries and FF files (in GROMACS format) for HA chains of arbitrary length (ZIP)
